# Hydroxyurea ameliorates atherosclerosis in ApoE^-/-^ mice by potentially modulating Niemann-Pick C1-like 1 protein through the gut microbiota

**DOI:** 10.7150/thno.76805

**Published:** 2022-11-14

**Authors:** Xin-Yu Yang, Hang Yu, Jie Fu, Hui-Hui Guo, Pei Han, Shu-Rong Ma, Li-Bin Pan, Zheng-Wei Zhang, Hui Xu, Jia-Chun Hu, Hao-Jian Zhang, Meng-Meng Bu, Xian-Feng Zhang, Wei Yang, Jing-Yue Wang, Jing-Yu Jin, Hui-Cong Zhang, Dong-Rui Li, Jin-Yue Lu, Yuan Lin, Jian-Dong Jiang, Qian Tong, Yan Wang

**Affiliations:** 1 The First Hospital of Jilin University, Changchun, 130021, China.; 2 State Key Laboratory of Bioactive Substance and Function of Natural Medicines, Institute of Materia Medica, Chinese Academy of Medical Sciences/Peking Union Medical College, Beijing 100050, China.

**Keywords:** Hydroxyurea, Atherosclerosis, Gut microbiota, Lipid metabolism, NPC1L1

## Abstract

**Rationale:** The efficacy and mechanism of hydroxyurea in the treatment of atherosclerosis have rarely been reported. The goal of this study was to investigate the efficacy of hydroxyurea in high-fat diet-fed ApoE^-/-^ mice against atherosclerosis and examine the possible mechanism underlying treatment outcomes.

**Methods:** ApoE^-/-^ mice were fed a high-fat diet for 1 month and then administered hydroxyurea by gavage continuously for 2 months. Aortic root hematoxylin-eosin (H&E) staining and oil red O staining were used to verify the efficacy of hydroxyurea; biochemical methods and ELISA were used to detect changes in relevant metabolites in serum. 16S rRNA was used to detect composition changes in the intestinal bacterial community of animals after treatment with hydroxyurea. Metabolomics methods were used to identify fecal metabolites and their changes. Immunohistochemical staining and ELISA were used for the localization and quantification of intestinal NPC1L1.

**Results:** We showed that aortic root HE staining and oil red O staining determined the therapeutic efficacy of hydroxyurea in the treatment of atherosclerosis in high-fat diet-fed ApoE^-/-^ mice. Serological tests verified the ability of hydroxyurea to lower total serum cholesterol and LDL cholesterol. The gut microbiota was significantly altered after HU treatment and was significantly different from that after antiplatelet and statin therapy. Meanwhile, a metabolomic study revealed that metabolites, including stearic acid, palmitic acid and cholesterol, were significantly enriched in mouse feces. Further histological and ELISAs verified that the protein responsible for intestinal absorption of cholesterol in mice, NPC1L1, was significantly reduced after hydroxyurea treatment.

**Conclusions:** In high-fat diet-fed ApoE^-/-^ mice, hydroxyurea effectively treated atherosclerosis, lowered serum cholesterol, modulated the gut microbiota at multiple levels and affected cholesterol absorption by reducing NPC1L1 in small intestinal epithelial cells.

## Introduction

Atherosclerotic disease is the most common chronic disease worldwide 1. Currently, it is considered to be a complex metabolic disorder, and its major risk factors include high blood pressure, high blood lipids, heavy smoking, diabetes, obesity and genetic factors 2. During the development of atherosclerosis, intimal injury, inflammatory response, oxidative stress, lipid infiltration, platelet activation, and vascular smooth muscle cell activation may occur 3. In addition to these classic risk factors and pathogenic processes, novel targets, such as the gut microbiota, have been confirmed to be associated with the development of atherosclerosis 4.

Despite the complicated pathogenesis, oral drug therapy for atherosclerosis mainly focuses on antiplatelet areas, regulating lipid metabolism and improving endothelial function 5,6. Currently, the combination of antiplatelet and cholesterol-lowering drugs (mainly aspirin combined with statins) remains the most mainstream drug for the treatment of atherosclerotic diseases 7,8. In addition, ezetimibe, a Niemann-Pick C1-like 1 (NPC1L1) inhibitor, is also widely used in clinical practice to lower circulating cholesterol by inhibiting the intestinal absorption of cholesterol through inhibition of the NPC1L1 receptor in small intestinal epithelial cells 9. Some other new cholesterol-lowering drugs, such as proprotein convertase subtilisin/kexin type 9 (PCSK9) inhibitors and bile acid chelators, have also been shown to have a role in the clinic 10,11. Another option for the treatment of atherosclerotic disease is interventional therapy, where some antiproliferative drugs have been used. For example, rapamycin and its derivatives are widely used as coatings of drug-eluting stents and drug-coated balloons for intervention 12. Other antiproliferative drugs, such as methotrexate 13,14 and sulfur purine 15, are also reported to be effective for atherosclerosis. However, the role of these drugs in atherosclerosis is limited to interventional therapy, and no oral antiproliferative drugs have been approved for clinical use in atherosclerosis.

Above all, the development of oral antiatherosclerotic drugs has mainly focused on lowering blood lipids, especially low-density lipoprotein cholesterol. In our clinical work, we discovered that a patient with acute myocardial infarction combined with first detected primary thrombocytosis showed a significant improvement in coronary atherosclerosis after two years of hydroxyurea treatment. Hydroxyurea (HU) is an antitumor drug that is administered orally. During the treatment period, the patient emphasized that the history of chronic diarrhea had also improved. This finding aroused our interest in the antiatherosclerotic effect and mechanisms of hydroxyurea.

Since HU will inevitably contact intestinal bacteria, our research focused on the gut microbiota. The gut microbiota refers to the symbiotic microbial species in the gastrointestinal tract 16. The human gut harbors more than 10^14^ microbial cells, including bacteria, fungi, archaea and viruses, which are an important and complex part of our healthy physiological ecosystem with bacterial populations 17. The gut microbiota have been shown to be associated with various systemic diseases, including neurological diseases, cardiovascular diseases and metabolic diseases 18-21. The gut microbiota have been shown to significantly affect lipid metabolism and the development of atherosclerosis 4,22,23. Previously, we demonstrated that the gut microbiota plays an important role in the treatment of hyperlipidemia, that hyperlipidemia can lead to changes in the diversity of the gut microbiota and that such changes can be modified by drug treatment 24,25.

In this study, we aimed to demonstrate that oral administration of HU is effective in the treatment of atherosclerosis in ApoE knockout (ApoE^-/-^) mice. Using 16S ribosomal RNA, nontargeted metabolomics and histological techniques, we investigated whether the therapeutic mechanism of HU is related to the gut microbiota and lipid metabolism. Therefore, we confirmed whether HU can be used as a potential clinical drug for the treatment of atherosclerosis.

## Materials and Methods

### Chemicals and Reagents

Atorvastatin calcium, aspirin and hydroxyurea (purity>98%) for animals were purchased from Solarbio Biotechnology Co., Ltd. (Beijing, China). Clopidogrel bisulfate (purity> 98%) was provided by the TCI Chemical Industry Development Co., Ltd. (Shanghai, China). Berberine (98%) was obtained from J&K Scientific Co., Ltd. The saturated oil red O solution and OCT frozen section embedding agent were purchased from Solarbio Biotechnology. Co., Ltd. (Beijing, China). Deionized distilled water was obtained from Hangzhou Wahaha Group Co., Ltd. (Hangzhou, China). Chromatography-grade methanol and isopropanol were obtained from Thermo Fisher Scientific, Co., Ltd. (Fair Lawn, NJ, United States). Other chromatographic reagents were obtained from Sinopharm Chemical Reagent Co., Ltd. (Beijing, China). Serum glucose (Glu), triglycerides (TG), total cholesterol (CHO) and low-density lipoprotein cholesterol (LDL-C), alanine transaminase (ALT), aspartate aminotransferase (AST) and creatinine kits were purchased from Biosino BioTechnology & Science Inc. Interleukin-1β (IL-1β), interleukin-6 (IL-6), tumor necrosis factor-ɑ (TNF-ɑ), oxidized low density lipoprotein (ox-LDL) and NPC1L1 kits were obtained from Nanjing Jiancheng Bioengineering Institute.

### Animals

Seven 8-week-old wild-type (C57BL/6) and 42 ApoE^-/-^ mice (8 weeks) were acquired from Beijing HFK Bioscience Co., Ltd. The animals were housed in SPF-grade rooms with a temperature of 22-24 °C, humidity of 45%, and a 12-h light/dark cycle (light time from 8:00 to 20:00). All mice had free access to food and water during the treatment. All experiments were conducted in accordance with institutional and ethics guidelines and were approved by the Laboratories Institutional Animal Care and Use Committee of the Chinese Academy of Medical Sciences and Peking Union Medical College (No. 00001020).

### Grouping and medication

The grouping rules were as follows: wild-type C57 mice were used in the normal control groups, and ApoE^-/-^ mice were used in the atherosclerosis model and medication groups. After acclimatization with a normal diet for a week, all ApoE^-/-^ mice were randomly assigned to 7 groups, including the model group and various drug administration groups. The normal control groups were fed a normal diet, and the model groups and medication groups were fed a high-fat diet (HFD): high-fat rodent diet with 1.25% cholesterol) for 4 weeks. Then, all the groups were treated with saline or drugs for 8 weeks for the efficacy study. Fasting serum, feces, intestine, liver and aortic vessels were obtained after the experiment for further study.

**Figure 1:**
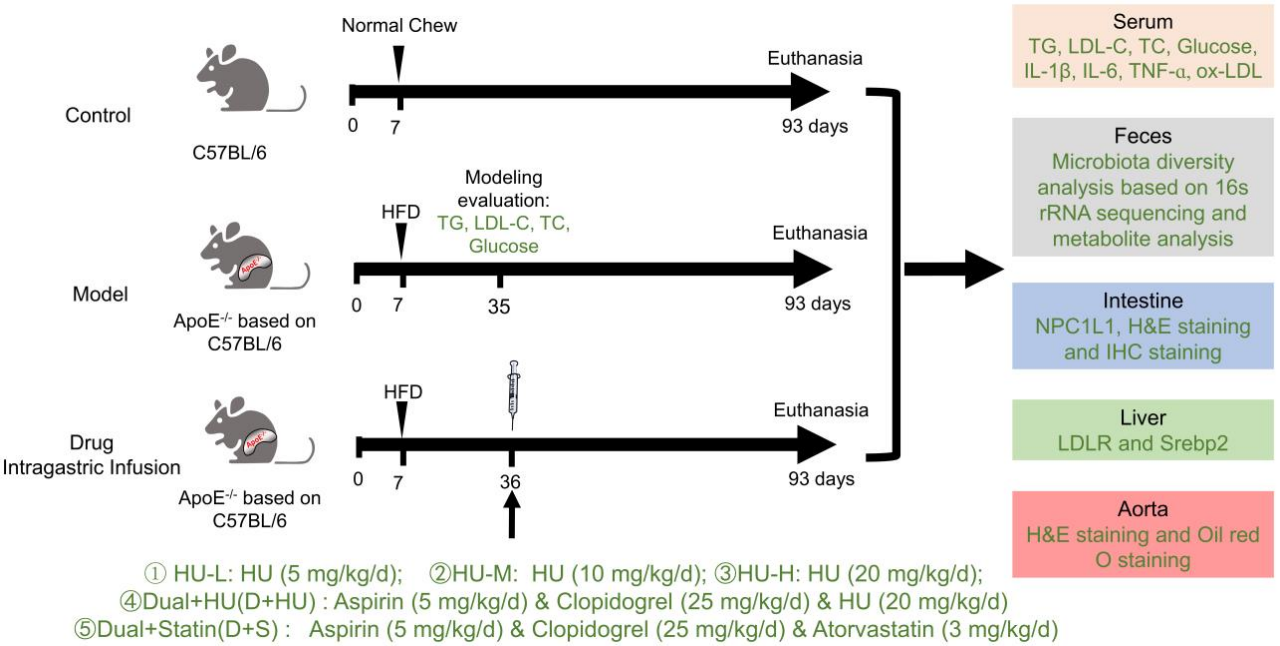
** Experimental design and general condition monitoring during the experimental period** (TG: triglycerides; LDL-C: low-density lipoprotein cholesterol; TC: total cholesterol; Glu: glucose; IL-1β: interleukin-1β; IL-6, interleukin-6; TNF-ɑ, tumor necrosis factor-ɑ; ox-LDL: oxidatively modified low-density lipoprotein; LDL-C: low-density lipoprotein cholesterol; LDLR: low density lipoprotein receptor; SREBP2, Sterol-regulatory element binding proteins 2; HFD: high fat diet; H&E: hematoxylin-eosin).

All the mice were separated into 7 groups (n = 7, Figure 1): (1) The normal control group (Control): normal diet and normal saline (gavage, 0.2 mL); (2) Atherosclerosis model group (Model): high-fat diet and normal saline (gavage, 0.2 mL); (3) Low-dose HU therapy group (HU-L): high-fat diet and HU (gavage, 5 mg/kg/day); (4) Medium-dose HU therapy group (HU-M): high-fat diet and HU (gavage, 10 mg/kg/day); (5) High-dose HU therapy group (HU-H): high-fat diet and HU (gavage, 20 mg/kg/day); (6) Combined medicine group (D+HU): high-fat diet, aspirin (gavage, 5 mg/kg/day), clopidogrel bisulfate (gavage, 25 mg/kg/day), and HU (gavage, 20 mg/kg/day); (7) Positive medicine group (D+S): high-fat diet, aspirin (gavage, 5 mg/kg/day), clopidogrel bisulfate (gavage, 25 mg/kg/day).

### Sample collecting

After 8 weeks of medication, mice faeces were collected for 16S rRNA gene sequencing and metabolomics analysis. After the mice were anesthetized, serum samples were collected for testing of blood lipids, glucose, cytokine factors, liver function and kidney function. Basal segments of mice heart were immediately collected and fixed in 4% paraformaldehyde for further staining. Jejunal segments of mice intestine were collected and fixed in 4% paraformaldehyde. The origin of jejunum is localized 20 mm from the pylorus in mice. The initial 5 mm of each mouse jejunal segment was used for immunohistochemistry and the next 10 mm was used for ELISA measurements of NPC1L1.

### Hematoxylin-eosin (H&E) staining

The aortic root and intestine tissue were fixed in 4% paraformaldehyde for 24 hours after being collected and dehydrated for 16 hours by an automatic dehydrator. After routine embedding with paraffin in an automatic embedding machine, the tissue was cut into sections (5 µm). Then, the sections were placed in an oven (60 °C) for 1 hour and dewaxed with xylene, ethanol and water. The sections were further stained with hematoxylin for 10 minutes and placed under running water to remove residual color. Then, 5% acetic acid was used to differentiate the cells, which were then stained with eosin. Finally, the sections were sealed with neutral gelatin after drying, and images were captured by the microscope system.

### Oil red O staining

The aorta root that attached to the heart was used for cross-sections. The collected aortic tissue was fixed in 4% paraformaldehyde for 24 hours and dehydrated overnight. Then, the tissue was embedded in the embedding agent OCT to prepare frozen slices (5 µm) at -20 °C. A saturated oil red O solution dissolved in isopropanol was mixed with distilled water at a ratio of 3:2 (*v:v*). After filtration, the frozen slices were stained in the diluted oil red O working solution at room temperature for 6 hours. Then, the slices were rinsed in 60% isopropanol-water solution 3 times and once in running water. The slices were restrained in hematoxylin solution for 1-2 min and washed with running water. The dyed slices were sealed with neutral gelatin, photographed and recorded by a microscope system (Leica, Germany). The corrected plaque areas were calculated as follows: Area of plaques/Area of vascular × 100% in the corresponding cross section. Lesion size was measured on digital microphotographs using ImageJ software (NIH, Bethesda, MD). The statistical results of the corrected plaque area were the average values of the plaque area obtained by oil red O staining and H&E staining of each mouse.

### Immunohistochemical staining

Intestinal samples were embedded in paraffin, sliced into 4-µm-thick sections for further staining and incubated with anti-NPC1L1 (Novus, USA) antibodies overnight at 4 °C. Then, the tissues were washed three times with PBS, incubated with goat anti-rabbit secondary antibody (Servicebio, China) for 50 min at 37 °C, and counterstained with hematoxylin. Immunopositive cells in the tissues were observed and scanned with a light microscope (Nikon, Tokyo, Japan).

### Serum and plasma analysis

Serum and plasma samples were collected from the fundus venous plexus, and the tubes for collecting plasma were pretreated with heparin sodium. Glu, TG, CHO, LDL-C, ALT, AST, creatinine, IL-1β, IL-6, TNF-ɑ and ox-LDL were measured according to the instructions of the kit.

### Microbial diversity analysis

Microbial DNA from fecal samples was isolated using the E.Z.N.A.® soil DNA kit (Omega Bio-Tek, USA) according to the manufacturer's instructions. The V3-V4 region of the bacterial 16S rRNA gene was amplified with the primer pairs 338F (5'-ACTCCTACGGGAGGCAGCAG-3') and 806R (5'-GGACTACHVGGGTWTCTAAT-3'). The PCR product was then extracted from a 2% agarose gel and purified using the AxyPrep DNA Gel Extraction kit (Axygen Biosciences, USA). The purified amplicons were then subjected to Illumina MiSeq sequencing analysis.

The 16S rRNA sequencing data were analyzed with the Quantitative Insights into Microbial Ecology platform (V.1.9.1). Operational taxonomic units (OTUs) with similarity over 97% were selected for taxonomy identification with the Greengenes database (V.13.8).

### Fecal Metabolomics analysis

Fecal samples were immediately stored at -80 °C until analysis. For fecal metabolomics analysis, sample preparation was slightly modified based on our previous protocol 26. Briefly, 300 µL of methanol containing 10 µg/mL succinic-d4 acid, serving as an internal standard, was added to 100 µL of fecal solution for protein precipitation. After centrifuging for 10 min at 10,000×*g* at 4 °C, 100 µL of supernatant was transferred to a new vial. Then, all the samples were dried under a stream of N_2_. Subsequently, samples were reconstituted in a 1:1 (*v/v*) solution of acetonitrile and the derivatizing agent BSTFA (1% tetramethylsilane, Sigma-Aldrich). The derivatization process was operated at 70 °C for 1 h. Samples were centrifuged again and pipetted into new HPLC vials with inserts. Then, metabolomics analysis was performed on a Shimadzu GC-2010 Plus gas chromatography system coupled to a GCMS-QP2020 SE single quadruple mass spectrometer (Shimadzu, Kyoto, Japan). One microliter of sample was injected in split mode with a split ratio of 1:120. The initial oven temperature was maintained at 60 °C for 5 min and then raised to 300 °C at a rate of 10 °C/min, holding for 5 min. The temperatures for injection, ion source and interface were set at 280, 200 and 300 °C, respectively. Mass data were collected in SCAN mode (*m/z* 50-600 Da) with an event time of 0.2 s. Quality control (QC) samples made from pooled corresponding samples were injected periodically.

### Statistical analyses

Database management and statistical analyses were performed with PRISM version 8.2.0 (GraphPad Software Inc., USA), SIMCA version 14 (MKS Umetrics AB, Sweden) and R 1.4.1.

### Metabolomics data statistical analysis

Method of metabolomics data statistical analysis were adapted from our previous protocol 26,27. The GC-MS data obtained were processed (peak picking and alignment) within the “XCMS” package in R Studio. After being normalized separately in R, features with a relative standard deviation < 30% in QC samples and present in 70% of the samples were included for subsequent multivariate analysis. Following log transformation and Pareto scaling, the data were subjected to orthogonal partial least squares-discriminant analysis (OPLS-DA). An S-plot derived from this OPLS-DA model was then applied to select features based on covariance P^1^ and correlation P(corr) values (P^1^ > 0.05, P(corr) > 0.7, or P^1^ < -0.05, P(corr) < -0.7). All multivariate analyses were conducted using SIMCA version 14 (MKS Umetrics AB, Sweden) 27. The most differentially expressed features selected were semiquantified in the raw data. Feature levels were expressed as ratios of peak areas to the peak areas of the internal standards. Identification was performed by comparing the fragmentation patterns of the metabolites detected with the spectra from the National Institute of Standards and Technology (NIST). After the raw data and NIST library were checked, metabolites with similarity indices over 85 were retained. Then, the peak areas of selected features were measured in the raw data (normalized to the internal standard) for univariate analysis. A one-way ANOVA with *P* < 0.05 was considered different metabolites. Finally, multiple comparisons were used. False Discovery Rate (FDR, Q = 0.05) approach was used for correction of multiple comparisons. Significance was assumed at a two-sided *P < 0.05, **P < 0.01, or ***P < 0.001.

### Experimental data statistical analysis

All values are expressed as mean ± standard error. GraphPad PRISM 5 (GraphPad Software Inc., USA) was used for statistical analysis. To determine the statistical difference between the two groups, a comparison of two groups was performed by a two-tailed unpaired t-test and Mann-Whitney U test, as appropriate. To determine the statistical differences between multiple groups, one-way analyses of variance (ANOVA) following multiple comparisons were used. False Discovery Rate (FDR, Q = 0.05) approach was used for correction of multiple comparisons. A linear regression model among group HU-L, HU-M and HU-H was applied to determine the dose-dependence. To investigate the association of each metabolite and bacterial strain with atherosclerosis biological variables, Spearman's correlation was applied. Significance was assumed at a two-sided *P < 0.05, **P < 0.01, or ***P < 0.001.

## Results

### HU decreased atherosclerosis plaque areas in ApoE^-/-^ mice

**Figure 2:**
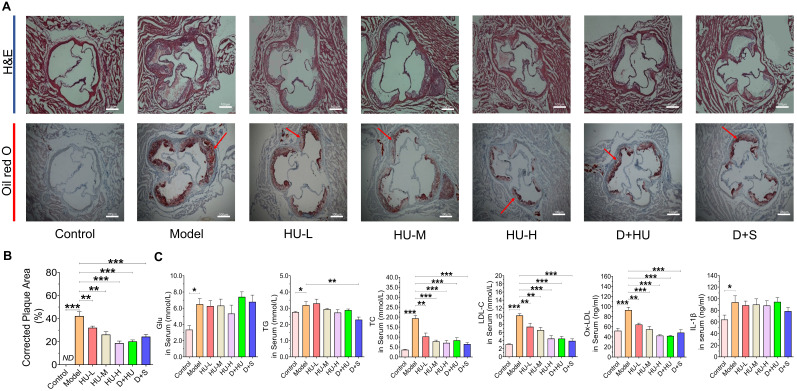
** Histological changes in aortic atherosclerotic plaques and changes in serum glucose and lipid metabolism and inflammatory parameters in various groups of mice. (A)** Aortic H&E staining and oil red O staining of each group (scale bars represent 100 um). The red arrow indicates atherosclerosis; **(B)** corrected plaque areas of atherosclerosis (%); **(C)** lipid, glucose and inflammatory factor levels after two months of drug administration. (n = 7, ^*^: compared to the model group, ^***^P < 0.001, ^**^ P < 0.01, ^*^ P < 0.05) (H&E: hematoxylin-eosin; Glu: glucose; IL-β: interleukin-β; TC: total cholesterol; LDL-C: low-density lipoprotein cholesterol; TG, total triglyceride).

ApoE^-/-^ mice are the most widely used animal models in evaluating atherosclerotic drugs 28. Herein, to evaluate the anti-atherosclerotic effects of HU, a high-fat diet-induced atherosclerotic model in ApoE^-/-^ mice was used. No significant differences in body weight or food intake were observed among all groups during the experiment (Supplementary material, Figure S1), and one mouse in the HU-M group died during the experiment due to the gavage operation. After 8 weeks of HU therapy, Figure 2a shows that the aortic vessels in the atherosclerosis model group presented a larger area of lipid-containing lesions and obvious plaques than those in the normal control group after 12 weeks of modeling according to aortic root oil red O staining. The areas of plaques showed a significant decrease in animals in the HU groups when compared to the plaques in animals in the model group (n = 7, **P < 0.01, ***P < 0.001, Figure 2A-B), and the changes were dose-dependent (R^2^ = 0.599, *P* < 0.001). Moreover, the results suggested that the anti-atherosclerotic effect of high-dose HU showed no significant difference from the effect of dual antiplatelet plus statin therapy (n = 7, *P* = 0.17).

### HU regulated lipid metabolism in ApoE^-/-^ mice

Serum glucose content, TG content, TC content, LDL-C and ox-LDL content are important indicators to evaluate the risk of atherosclerosis 29. Additionally, inflammation plays an important role in the development of atherosclerosis 30. Therefore, we investigated the levels of these markers in mouse serum after two months of drug administration. In Figure 2C, a significant decrease in TC, LDL-C and ox-LDL is observed in the HU therapy groups compared to the model group (n = 7, *P < 0.05, **P < 0.01, ***P < 0.001, Figure 2C). The ability of HU to lower LDL-C and ox-LDL was dose-dependent (R^2^ = 0.397, *P* < 0.01 for LDL-C and R^2^ = 0.588, *P* < 0.001 for ox-LDL). Meanwhile, HU did not exhibit the ability to lower serum glucose, IL-1β, IL-6 or TNF-ɑ (Figure 2C and Supplementary material, Figure S2). These modifications in lipid metabolism provide insights for further exploration of the anti-atherosclerotic mechanism of HU.

### HU demonstrated good drug safety during treatment

Since HU is an antitumor agent and possesses cytotoxic properties, its safety in the treatment of atherosclerosis requires intensive attention. Therefore, we investigated the indicators of hepatotoxicity and nephrotoxicity after two months of drug administration. Serum ALT, AST and creatinine levels were tested. A significant increase in ALT is observed in the D+S group compared to the model group (n = 7, *P < 0.05, Supplementary material, Figure S3A), while HU exhibited no significant effect on AST and ALT levels. Meanwhile, HFD modeling increased serum creatinine levels (n = 7, **P < 0.01, Supplementary material, Figure S3C), but drug treatment did not increase them further, suggesting that neither significant hepatotoxicity nor renal toxicity occurred after treatment with HU at this effective dose.

### HU altered ecological diversity of the fecal microbiota

**Figure 3:**
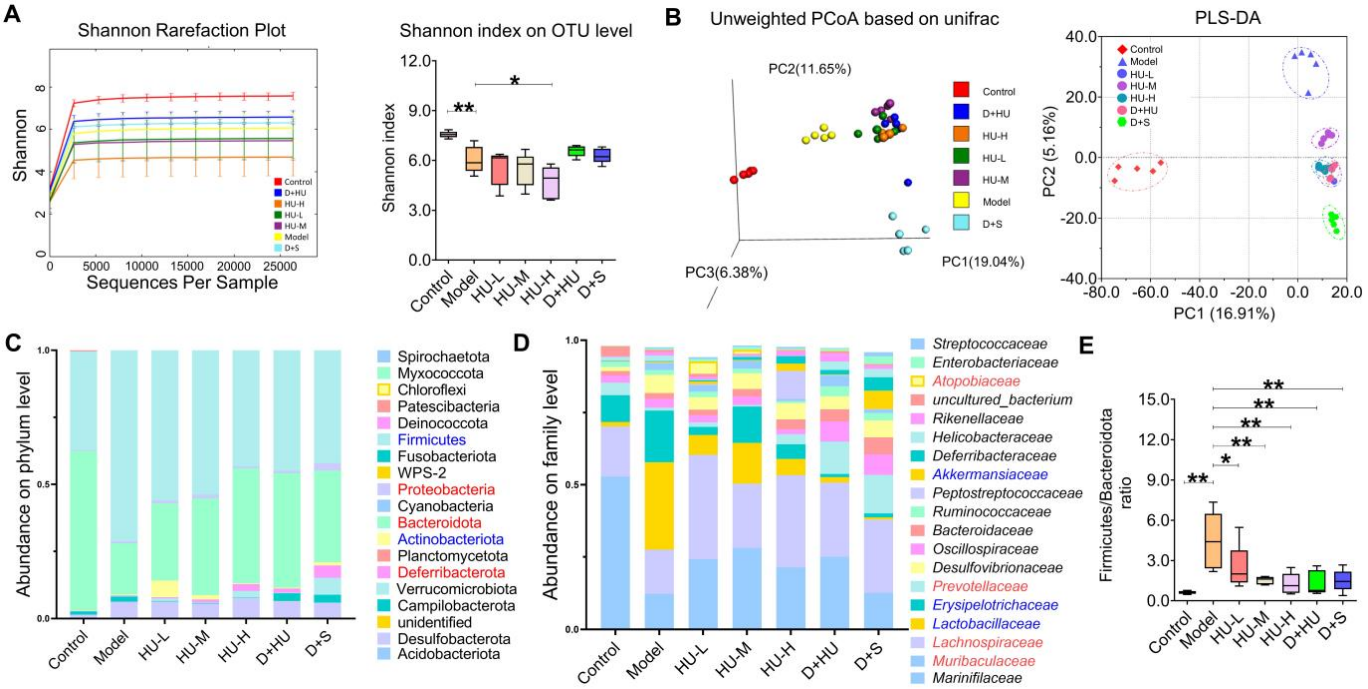
** Microbiota diversity analysis at different levels. (A)** Multisample Shannon-Wiener curves and Shannon index of each group at the OTU level; **(B)** PCoA and PLS-DA model summarizing the distribution of samples; **(C)** community analysis based on the phylum level; **(D)** community analysis based on the family level; **(E)** Firmicutes/Bacteroidetes ratio. (n = 5, ^*^: compared to the model group,^ **^P < 0.01, ^*^P < 0.05) (OTU: operational taxonomic unit; PCoA: principal co-ordinates analysis; PLS-DA: partial least-squares discrimination analysis).

Gut microbiota are now thought to influence the development of atherosclerosis at multiple targets, such as lipid metabolism and atherosclerosis 31. The fecal microbiome composition was analyzed to explore compositional differences and the extent to which the microbiome was altered in animals in different groups. Fecal samples were treated for 16S rRNA sequencing as explained above (n = 5). Figure 3a shows that the Shannon-Wiener curves tend to be flat, indicating that the test can reflect the majority of microbial information in the sample. The Shannon index showed a significant reduction in the ɑ-diversity of the gut microbiota in the high-dose group (n = 5, *P < 0.05, **P < 0.01, Figure 3A). The PCoA and PLS-DA results (Figure 3b) based on 4518 OTUs demonstrated clear separation among each group in terms of gut microbiota composition. Interestingly, distribution of groups containing HU treatment clustered together and were clearly separated from not only the model group but also the positive medication (D+S) group, suggesting that gut microbiota alterations after HU treatment may follow a different pattern than those of the antiplatelet plus statin groups. To examine the specific changes in microbiota in ApoE^-/-^ mice, we assessed the relative abundance of taxa among each group at different levels. Figure 3c and 3d demonstrated the changes among groups at both the phylum and family levels. Firmicutes and Bacteroidota were the predominant phyla, and the ratio of Firmicutes to Bacteroidota (F/B) was higher in animals in the model group than in animals in the control group (**P < 0.01, Figure 3e). This change was corrected by both HU treatment and D+S treatment (*P < 0.05, **P < 0.01, Figure 3e). At the family level, we identified a tendency for the *Lactobacillaceae* family to decrease and the *Lachnospiraceae* family to increase in abundance after HU treatment (*P < 0.05, Figure 3d).

**Figure 4:**
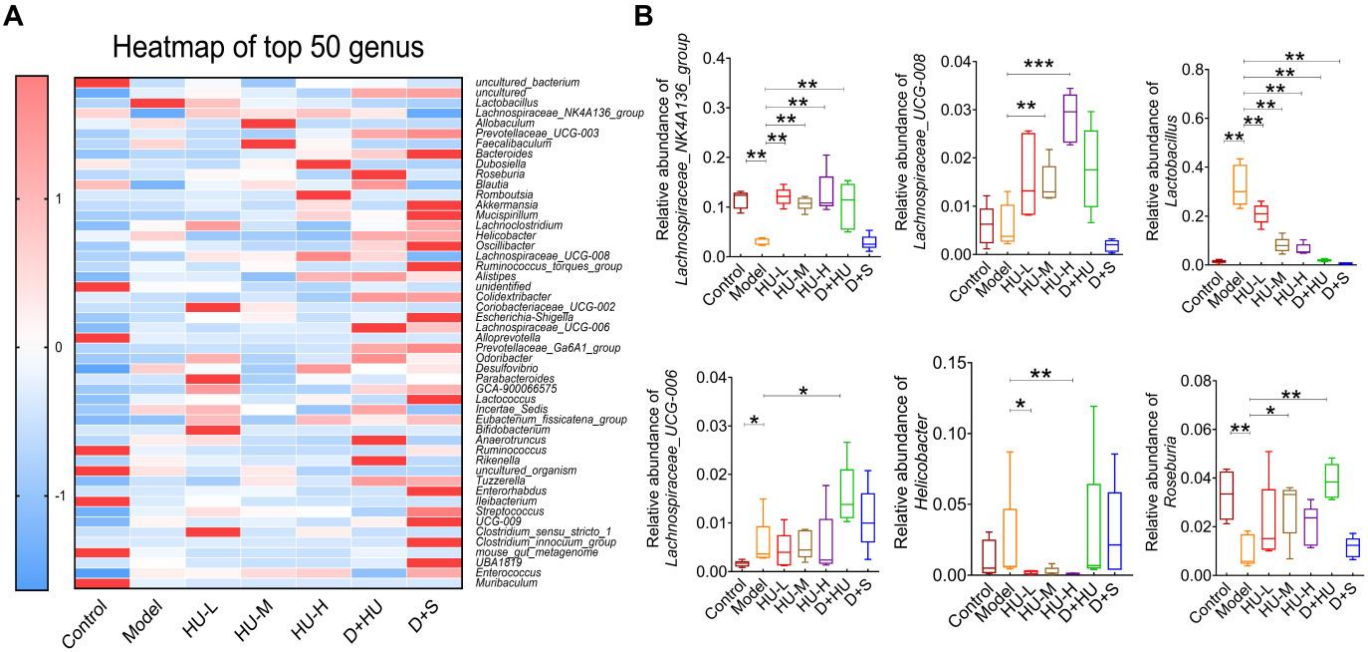
** Microbiota diversity analysis at the genus level. (A)** Heatmap showing the top 50 genera; **(B)** analysis of bacterial strains at the genus level. (n = 5, ^*^: compared to the model group, ^***^P < 0.001, ^**^P < 0.01, ^*^P < 0.05).

At the genus level, the heatmap of the top 50 genera clearly illustrates changes in gut microbiota composition in each group (Figure 4a). Using the Disbiome database 32, we retrieved the association of these genera with the development of hyperlipidemia or atherosclerotic diseases in human (Supplementary Table 1). With the available evidence, we identified 6 genera that had significantly altered abundance before and after HU treatment and were closely associated with atherosclerotic diseases, namely, *Lactobacillus*,* Lachnospiraceae_NK4A136_group*,* Lachnospiraceae_UCG-008*,* Lachnospiraceae_UCG-006*,* Roseburia* and *Helicobacter*. Overall, the abundance of *Lactobacillus* and *Helicobacter* was significantly decreased after HU therapy (*P < 0.05, **P < 0.01, Figure 4B), while the abundance of *Lachnospiraceae_NK4A136_group*,* Lachnospiraceae_UCG-008*,* Lachnospiraceae_UCG-006* and *Roseburia*, which all belong to the *Lachnospiraceae* family, was significantly increased (*P < 0.05, **P < 0.01, Figure 4B).

### Alterations in the fecal metabolic profile of ApoE^-/-^ mice

**Figure 5:**
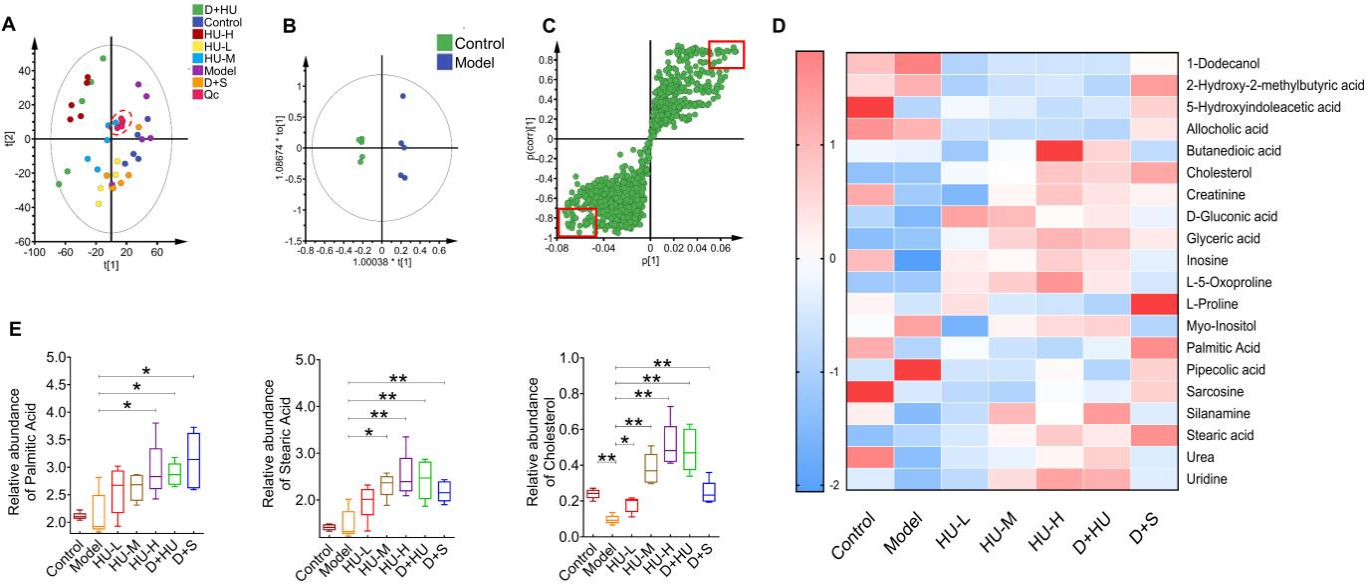
** Metabolic profiling of fecal samples from each group. (A)** 2D and 3D examples of PCA score plots summarizing the distribution of QCs and samples.** (B)** An OPLS-DA model showing the group separation between the control group and the model group. **(C)** Corresponding S-plot for feature selection. Each dot represents a feature, and the dots located in the upper right and lower left areas are the selected dots based on the criteria stated in the Methods section. **(D)** Heatmap showing metabolic features among each individual. **(E)** Box plot showing metabolic feature levels related to lipid metabolism and atherosclerosis among each group. Data represent the mean ± S.D. of 5 individuals in each group. (n = 5, ^*^: compared to the model group, ^***^P < 0.001, ^**^P < 0.01, ^*^P < 0.05) (PCA: principal component analysis; QC: quality control; OPLS-DA: orthogonal partial least squares-discriminant analysis).

To further analyze how intestinal bacteria affect serum glycolipid changes and eventually atherosclerosis, we used metabolomics analysis of fecal samples to gain insight into any metabolic deviation associated with glycolipid metabolism (n = 5). The principal component analysis (PCA) plot showed that all QC samples clustered near the origin, indicating good reproducibility. Meanwhile, PCA also demonstrated clear separations in plasma metabolic profiles among each group, as samples of each group clustered in different areas (Figure 5A). OPLS-DA models further confirmed the separation between every pair of groups, indicating a biochemical alteration (Figure 5B, Supplementary file, Figure S4). To identify which metabolic feature was the strongest discriminator among the groups, the corresponding correlation S-plots (Figure 5C) were used to generate a list of features of interest on the criteria stated in the Methods section. Ultimately, 20 metabolites were identified as features of interest.

Heatmap showed 20 features were identified from the analysis of significant differences, namely, 1-dodecanol, 2-hydroxy-2-methylbutyric acid, 5-hydroxyindoleacetic acid, allocholic acid, butanedioic acid, cholesterol, creatinine, d-gluconic acid, glyceric acid, inosine, l-5-oxoproline, l-proline, myo-inositol, palmitic acid, pipecolic acid, sarcosine, silanamine, stearic acid, urea, and uridine (Figure 5D). Among these metabolites, we identified three metabolites related to lipid metabolism and atherosclerosis, namely, stearic acid, palmitic acid and cholesterol (Figure 5E). Stearic acid and palmitic acid are both saturated fatty acids 33,34. HU and positive medicine treatment both significantly increased the concentration of saturated fatty acids in feces (*P < 0.05, **P < 0.01, Figure 5E). Fecal cholesterol concentrations were significantly reduced in the model group and increased approximately 5-fold in the high-dose group (*P < 0.05, **P < 0.01, Figure 5E). In conclusion, fecal metabolomics suggests that cholesterol and specific saturated fatty acids are significantly aggregated in the feces after HU treatment.

### HU altered the abundance of NPC1L1 in the small intestine of ApoE^-/-^ mice

**Figure 6:**
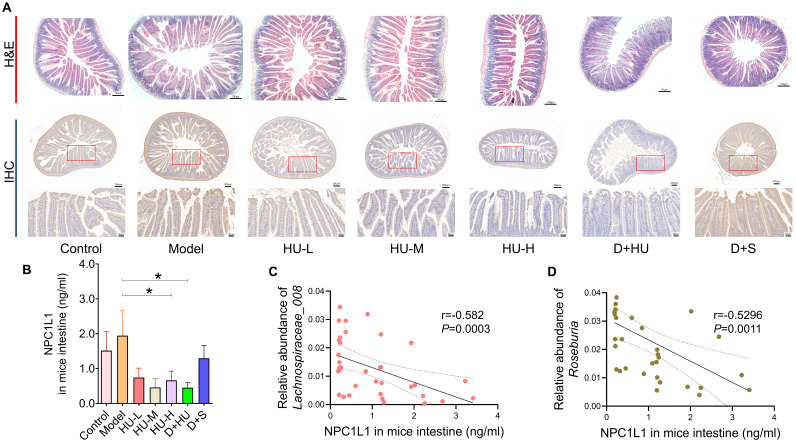
** NPC1L1 alteration and correlation with intestinal bacteria between groups. (A)** Representative H&E staining (scale bars represent 100 µm) and immunohistochemistry (scale bars represent 200 µm in the upper row and 50 µm in the lower row) of the small intestine of mice using an NPC1L1 antibody. **(B)** NPC1L1 levels in each group. **(C-D)** Correlation analysis between NPC1L1 and gut bacterial strains using Spearman's correlation (n = 5,^ *^: compared to the model group, ^*^P < 0.05) (NPC1L1: Niemann-Pick C1-like 1; H&E: hematoxylin-eosin; IHC: immunohistochemistry).

Based on the accumulation of fecal cholesterol and the reduction in plasma TC and LDL-C after HU treatment, we considered that HU might play a role in reducing circulating cholesterol and antiatherosclerosis by inhibiting cholesterol absorption from the digestive tract. NPC1L1 is an essential protein for cholesterol absorption 35. Figure 6A-B show immunohistochemistry and ELISA data that confirm that NPC1L1 expression in mouse small intestinal epithelial cells was significantly and dose-dependently reduced with HU treatment (n = 7, Figure 6C, *P < 0.05). However, the positive control did not alter the NPC1L1 concentration. In Figure 6D, Spearman's correlation coefficient was calculated between bacterial genera and NPC1L1, which showed that both *Roseburia* and *Lachnospiraceae_UCG-008* were negatively correlated with the expression of NPC1L1 (r = -0.582 for *Roseburia* and r = -0.5296 for *Lachnospiraceae_UCG-008*). Meanwhile, there were no significant changes in terms of hepatic sterol-regulatory element binding protein 2 (SREBP2) levels and low-density lipoprotein receptor (LDLR) levels among each HU therapy group (Supplementary file, Figure S5).

## Discussion

The study of antiatherosclerotic drugs has always been a topical issue in cardiovascular disease. In this study, we report that HU, a cytotoxic drug, can treat atherosclerosis through potential effects on intestinal cholesterol absorption by modulating the gut microbiota. HU is a nonalkylated cytotoxic drug that has been widely used in the treatment of malignant tumors or viral infections. It has been reported to be a nucleoside diphosphate reductase inhibitor that can block the reduction of nucleotides to deoxynucleotides, interfere with purine and pyrimidine base biosynthesis, and selectively block DNA synthesis. In 2009, Gallaugher reported that a high dose of hydroxyurea reduced the degree of aortic atherosclerosis in a secondary injury rabbit model of atherosclerosis 36. This study confirmed the potential antiatherosclerotic effect of HU, but the mechanism was not further explored.

In fact, it is frequently reported that cytotoxic drugs such as HU exhibit antiatherosclerotic effects. For example, docetaxel is reported to be effective in atherosclerosis by promoting HDL biogenesis 37, while resveratrol and cyclophosphamide have similarly demonstrated antiatherosclerotic effects through interactions with multiple molecular targets of diverse intracellular pathways 38,39. Most of these cytotoxic drugs have a common denominator, exerting beneficial effects at low doses and cytotoxic effects at high doses. Likewise, many cytotoxic drugs have been shown to have a clear relationship with gut microbiota. Temozolomide, an alkylated prodrug commonly used as a chemotherapeutic agent for glioma patients, has been shown to alter the gut microbiota of glioma mice and exert antitumor effects by altering specific bacterial genera 40.In our study, we found that the effect of HU on the gut microbiota was based on its down-regulation of its β-diversity, and this effect, similar to that of antibiotics, suggests that HU may complete the reconstruction of the gut microbiota and exert its pharmacological effects by killing some bacteria.

The gut microbiota and its metabolites have been shown to significantly affect lipid metabolism and the development of atherosclerosis 4,41. 16S rRNA gene sequencing results revealed significant changes in gut microbiota before and after HU treatment. At the phylum level, we found that HU restored the Bacteroidetes/Firmicutes ratio disrupted by the HFD, which is considered a marker of obesity and abnormal lipid metabolism in many studies 42,43. Furthermore, the analysis demonstrated that HU modulated the composition of the gut microbiota at all levels in HFD-fed ApoE^-/-^ mice. At the family level, the abundance of *Lactobacillaceae* was also restored by HU treatment. The current study suggests a more contradictory role for *Lactobacillaceae* in atherosclerosis, which is commonly considered to be a probiotic and is protective against a variety of diseases and has been reported to be in reduced abundance in patients with acute coronary syndrome 44. Huang et al. 45 reported a spp. of *Lactobacillaceae*, *Lactobacillus acidophilus ATCC 4356*, prevented the progression of atherosclerosis in ApoE^-/-^ mice by inhibiting intestinal cholesterol absorption. However, Chen et al. 46 reported that Lactobacillus casei accelerated atherosclerosis in a mouse model of Kawasaki disease. In the Tampere Sudden Death Study, *Lactobacillaceae* was positively correlated with coronary atherosclerotic plaque area in patients, and the DNA of *Lactobacillaceae* could be amplified in the coronary plaques, which suggested gut bacterial translocation 47. Meanwhile, we identified a significant increase in the abundance of the *Lachnospiraceae* family after HU treatment. We identified four genera belonging to the *Lachnospiraceae* family, *Lachnospiraceae_NK4A136_group*,* Lachnospiraceae_UCG-008*,* Lachnospiraceae_UCG-006* and *Roseburia,* that showed increased abundance after HU treatment. The ability of the *Lachnospiraceae* family to regulate lipid metabolism has also been well documented 48,49. Wu et al. 50 found that *Roseburia* was the key bacterium for the antiatherosclerotic effect of berberine in ApoE^-/-^ mice. *Lachnospira_NK4A136_group* was reported to be responsible for the alleviation of hypercholesterolemia 51. Since these three genera belong to the short-chain fatty acid-producing bacteria, mechanisms by which these genera affect lipid metabolism have always focused on the improvement of lipid metabolism by short-chain fatty acids but lacked analysis of lipid metabolic pathways.

Fecal metabolomics analysis showed that fecal cholesterol differed significantly between groups, while serum TC showed a significant decrease after HU treatment. This led us to investigate whether HU affects the intestinal absorption of cholesterol. There are two major sources of free cholesterol in feces. The first, of course, is from food intake, while the other is from reverse cholesterol transport (RCT). RCT represents a pathway of the efflux of cholesterol from peripheral cells to extracellular cholesterol receptors, the transfer of cholesterol from the interstitial fluid to the plasma and liver, and the excretion of hepatic free cholesterol through the bile to the intestinal tract and finally to the feces. Dietary cholesterol is an important source of individual cholesterol, with approximately 1/5 of cholesterol being absorbed through the diet 52, and RCT has been shown to be closely related to atherosclerosis at several levels 53. In total, the gut acts as a gate for 1500 mg of cholesterol entering the intestinal lumen per day 54. Before being excreted in the feces, free cholesterol in the intestine is partially absorbed or reabsorbed through the small intestine. Thus, changes in fecal free cholesterol suggest changes in intestinal absorption of dietary cholesterol and RCT-derived cholesterol, thereby affecting circulating TC levels, LDL-C levels, and atherogenesis.

NPC1L1, a glycosylated protein located in the brush border enterocyte membrane, is responsible for almost 70% of cholesterol absorption in mice 55. NPC1L1 plays an important role in cholesterol metabolism and RCT 54,56. Ezetimibe, an inhibitor of NPC1L1, has exhibited promising cholesterol-lowering and antiatherosclerotic effects in humans and animals 57. It has been widely used in clinical practice and is recommended by guidelines for the management of dyslipidemias 58. Therefore, it is plausible that the lipid-lowering and antiatherosclerotic effects can be achieved by regulating the expression of NPC1L1, which HU exhibited in our experiment.

Meanwhile, a correlation between the gut microbiota and NPC1L1 was also observed. Haghika and Hazen et al. 59 reported that pseudo-germfree status exacerbates HFD-induced abnormal cholesterol metabolism and atherosclerosis in ApoE^-/-^ mice and that a specific metabolite of intestinal bacteria, propionic acid, could improve cholesterol metabolism and atherosclerosis by decreasing the expression of NPC1L1 through an immune-dependent regulatory mechanism. A correlation between NPC1L1 and *Lachnospiraceae_UCG-008* or *Roseburia* was also found in our correlation analysis, which provides us with the next direction to further explain the antiatherosclerotic mechanism of HU.

In addition to cholesterol, fecal metabolomics analysis also showed significant changes in metabolites, including palmitic acid and stearic acid. Coincidentally, these three substances were the main components of the high-fat diet in the experiment. Palmitic acid and stearic acid are both saturated fatty acids. It is widely accepted that elevated cholesterol and impaired metabolism are the main causes of atherosclerosis. Additionally, excess saturated fatty acids in food are an important cause of obesity 60. The bulk of the current evidence suggests that diets rich in saturated cholesterol promote cholesterol synthesis and increase circulating LDL-C, finally leading to atherosclerosis 61,62. Considering that there was no significant difference in feed intake among each group of mice during the experiment, we hypothesized that HU also affected the intestinal absorption of palmitic acid and stearic acid. However, the mechanism needs to be further explored.

In conclusion, we report the pharmacological effects of HU in the treatment of atherosclerosis in ApoE^-/-^ mice. This effect may depend on the effect of HU on the gut microbiota and small intestinal NPC1L1. However, whether this efficacy is of clinical application needs to be further investigated. Currently, no clinical studies have confirmed the efficacy of HU on atherosclerosis. In our finding, HU altered some specific genera of the mice gut microbiota. Coincidentally, these genera are also present in the human gut microbiota and have been shown to be associated with atherosclerosis 63-65, which were altered in a beneficial manner to improve atherosclerosis after HU treatment. Meanwhile, drugs targeting NPC1L1 on the small intestine, such as ezetimibe, have been widely used in the treatment of atherosclerosis 58. We therefore believe that HU has sufficient potential for clinical translation. Nevertheless, efficacy of HU needs to be validated at the clinical level and its mechanisms need to be further explored.

## Supplementary Material

Supplementary figures and table.

## Abbreviations

ApoE^-/-^: ApoE-knockout
F/B: the ratio of Firmicutes to Bacteroidota
Glu: glucose
H&E: hematoxylin-eosin
IHC: immunohistochemistry
HFD: high fat diet with 1.25% cholesterol
HU: hydroxyurea
IL-1β: interleukin-1β
IL-6: interleukin-6
TNF-α: tumor necrosis factor-ɑ
LDL-C: low-density lipoprotein cholesterol
LDLR: low density lipoprotein receptor
SREBP2: Sterol-regulatory element binding proteins 2
ALT: alanine transaminase
AST: aspartate aminotransferase
NIST: National Institute of Standards and Technology
NPC1L1: Niemann-Pick C1-like 1
OPLS-DA: orthogonal partial least squares-discriminant analysis
OTUs: operational taxonomic units
PCoA: principal co-ordinates analysis
PLS-DA: partial least-squares discrimination analysis
ox-LDL: oxidatively modified low-density lipoprotein
PCA: principal component analysis
PCSK9: proprotein convertase subtilisin/kexin type 9
QC: quality control
RCT: reverse cholesterol transport
SREBP2: sterol-regulatory element binding proteins 2
TC: total cholesterol
TG: triglycerides
16S rRNA: 16S ribosomal RNA
