# Supplementary material for: Hydroxyurea ameliorates atherosclerosis in ApoE-/- mice by potentially modulating Niemann-Pick C1-like 1 protein through the gut microbiota
Source: Theranostics. 2022 Nov 14;12(18):7775–87. doi: 10.7150/thno.76805 (PMC9706578; doi:10.7150/thno.76805)
Supplement: Supplementary file 1 — Supplementary figures and table. [file thnov12p7775s1.pdf]

Hydroxyurea ameliorates atherosclerosis in ApoE$^{-/-}$ mice by potentially modulating Niemann-Pick C1-like 1 protein through the gut microbiota

Xin-Yu Yang [a,b,1], Hang Yu [b,1], Jie Fu [b,1] , Hui-Hui Guo [b], Pei Han [b], Shu-Rong Ma [b], Li-Bin Pan [b], Zheng-Wei Zhang [b], Hui Xu [b], Jia-Chun Hu [b], Hao-Jian Zhang [b], Meng-Meng Bu [b], Xian-Feng Zhang [a], Wei Yang [a], Jing-Yue Wang [a], Jing-Yu Jin [a], Hui-Cong Zhang [a], Dong-Rui Li [a], Jin-Yue Lu [b], Yuan Lin [b,*], Jian-Dong Jiang [b,*], Qian Tong [a,*], Yan Wang [b,*]

[a] The First Hospital of Jilin University, Changchun, 130021, China
[b] State Key Laboratory of Bioactive Substance and Function of Natural Medicines, Institute of Materia Medica, Chinese Academy of Medical Sciences/Peking Union Medical College, Beijing 100050, China

[*] Correspondence to: Y. Wang (+86-10-63165238, wangyan@imm.ac.cn) or, Q. Tong (+86-13074337289, tongqian@jlu.edu.cn) or, J-D. Jiang (+86-10-63017906, jiang.jdong@163.com) or, L. Yuan (+86-13720009342, linyuan@imm.ac.cn)

[1] These authors made equal contribution to this work.

Supplementary information

Figure legends

Supplementary Figure 1: Body weight and food intake of mice during the experiment. **(A)** Weight change of mice of each group. **(B)** Changes in the food intake of mice in each group (n = 7).

Supplementary Figure 2：Plasma IL-6 and TNF-ɑ levels after two months of drug administration. (n = 7, IL-6: interleukin-6; TNF-ɑ: tumor necrosis factor-ɑ).

Supplementary Figure 3：Plasma ALT, AST and creatinine levels after two months of drug administration. (n = 7, *: compared to the model group, *P < 0.05, ALT: alanine transaminase, AST: aspartate aminotransferase).

Supplementary Figure 4：OPLS-DA model showing the group separation between the model group and the medication group. **(A)** OPLS-DA model of model group vs HU-L group. **(B)** OPLS-DA model of model group vs HU-M group. **(C)** OPLS-DA model of model group vs HU-H group. **(D)** OPLS-DA model of model group vs D+HU group (n = 5, OPLS-DA: orthogonal partial least squares-discriminant analysis).

Supplementary Figure 5: LDLR and Srebp2 levels in mouse livers. **(A)** Hepatic LDLR levels among each group (n = 7, *: compared to the model group, $^*P < 0.05$, ns: not significant). **(B)** Hepatic LDLR levels among each group (n = 7, *: compared to the model group, $^*P < 0.05$, LDLR: low density lipoprotein receptor, SREBP2: Sterol-regulatory element binding proteins 2).

Table legend

Supplementary Table 1: Differential bacteria associated with lipid metabolism and atherosclerotic diseases

Supplementary Figure 1

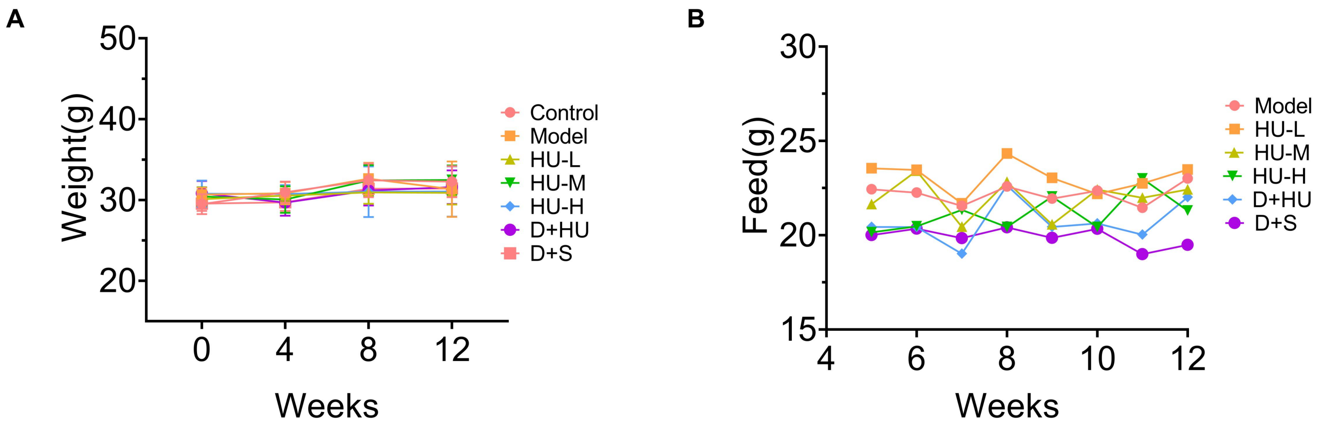

Supplementary Figure 2

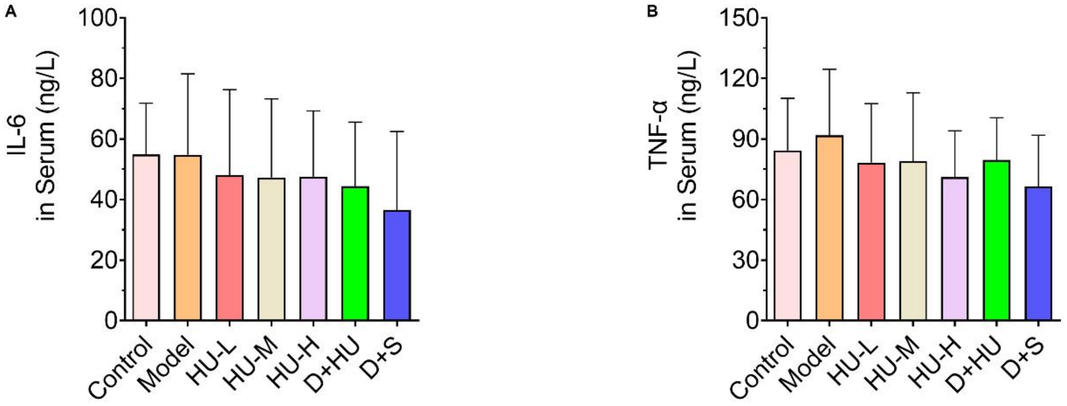

Supplementary Figure 3

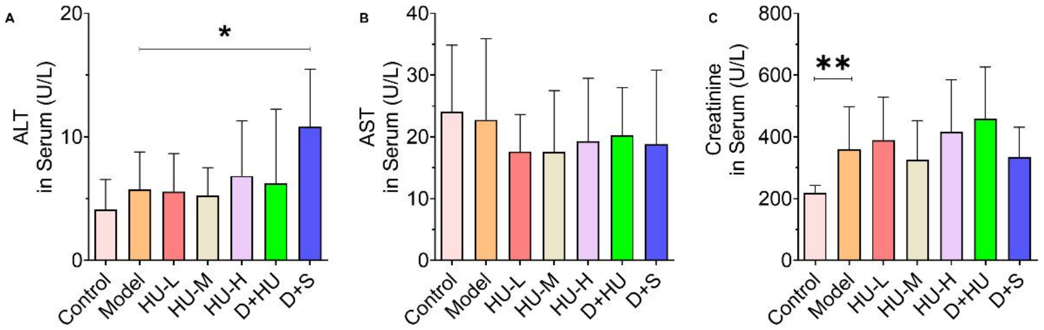

Supplementary Figure 4

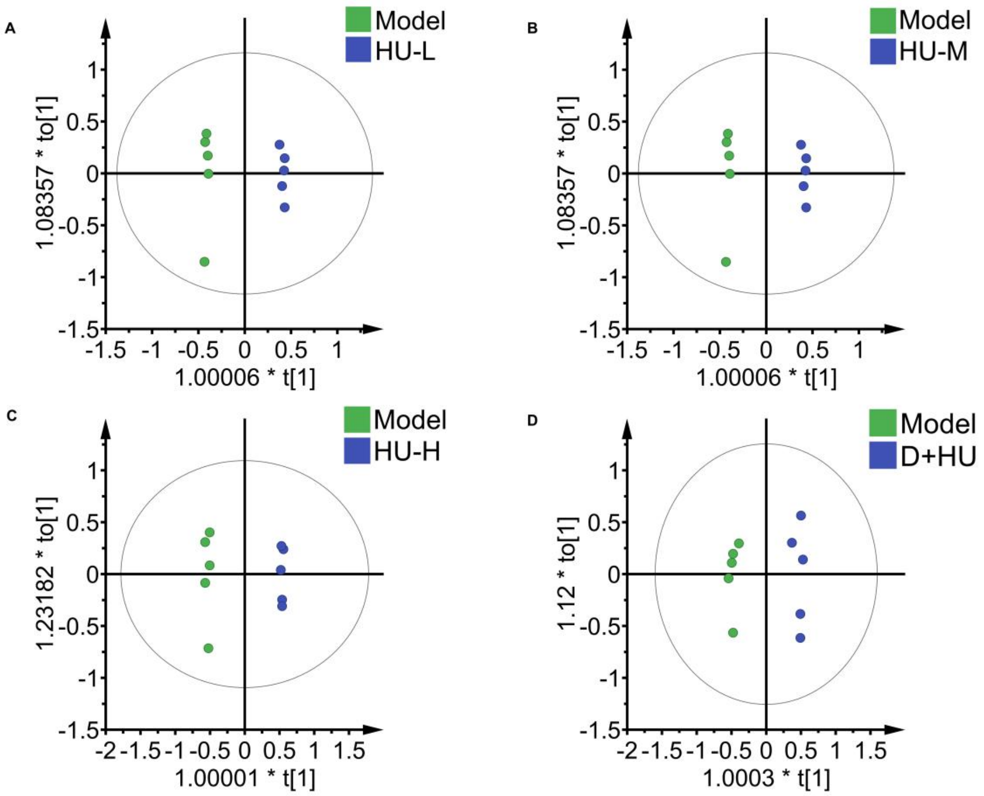

Supplementary Figure 5

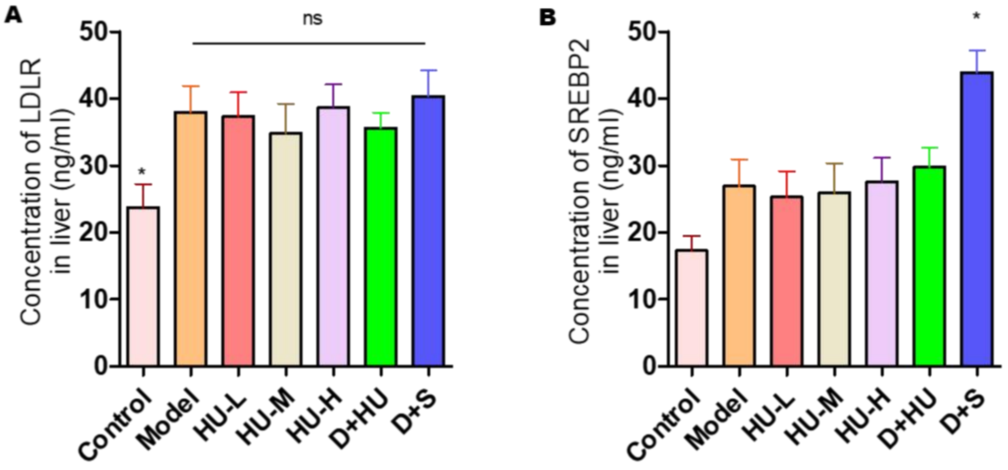

Supplementary Table 1 Differential bacteria associated with lipid metabolism and atherosclerotic diseases

Organism	Qualititative outcome	Diseases/Traits	Method	First author	Year of publication	Type	Host
Lactobacillus	Elevated	Coronary artery disease	16S rRNA sequencing	Liu Z[1]	2019	Faeces	Human
	Elevated	Coronary artery disease	16S rRNA sequencing	Zhu Q[2]	2018	Faeces	Human
Prevotella	Elevated	Coronary artery disease	16S rDNA pyrosequencing	Toya T[3]	2020	Faeces	Human
Lachnospiraceae	Reduced	Coronary artery disease	16S rRNA sequencing	Zhu Q[2]	2018	Faeces	Human
	Reduced	Coronary artery disease	16S rRNA sequencing	Liu H[4]	2019	Faeces	Human
Bacteroides	Reduced	Atherosclerosis	16S rRNA sequencing	Jie Z[5]	2017	Faeces	Human
	Elevated	Coronary artery disease	16S rRNA sequencing	Liu Z[1]	2019	Faeces	Human
	Reduced	Coronary artery disease	16S rRNA sequencing	Liu Z[1]	2019	Faeces	Human
	Reduced	Coronary artery disease	TRFLP	Emoto T[6]	2017	Faeces	Human
	Reduced	Hyperlipidemia	16S rRNA sequencing	Gargari G[7]	2018	Faeces	Human
Blautia	Reduced	Coronary artery disease	16S rRNA sequencing	Zheng Y-Y[8]	2020	Faeces	Human
Akkermansia	Elevated	Coronary artery disease	16S rRNA sequencing	Zheng Y-Y[8]	2020	Faeces	Human
	Elevated	Ischemic stroke	16S rRNA sequencing	Tan C[9]	2020	Faeces	Human
	Reduced	Hyperlipidemia	16S rRNA sequencing	Gargari G[7]	2018	Faeces	Human
Roseburia	Reduced	Coronary artery disease	16S rRNA sequencing	Zhu Q[2]	2018	Faeces	Human
	Reduced	Atherosclerosis	Shotgun sequencing	Karlsson F[10]	2012	Faeces	Human
	Reduced	Hyperlipidemia	16S rRNA sequencing	Gargari G[7]	2018	Faeces	Human
Odoribacter	Elevated	Coronary artery disease	16S rRNA sequencing	Zheng Y-Y[8]	2020	Faeces	Human
Desulfovibrio	Elevated	Coronary artery disease	16S rRNA sequencing	Zheng Y-Y[8]	2020	Faeces	Human
Parabacteroides	Elevated	Coronary artery disease	16S rRNA sequencing	Liu Z[1]	2019	Faeces	Human
	Elevated	Coronary artery disease	MiSeq sequencing	Kehrmann J[11]	2019	Faeces	Human

	Reduced	Coronary artery disease	16S rRNA sequencing	Zhang Y[12]	2019	Faeces	Human
Lactococcus	Elevated	Coronary artery disease	16S rRNA sequencing	Zhu Q[2]	2018	Faeces	Human
Ruminococcus	Elevated	Coronary artery disease	16S rDNA pyrosequencing	Toya T[3]	2020	Faeces	Human
	Elevated	Atherosclerosis	16S rRNA sequencing	Jie Z[5]	2017	Faeces	Human
Streptococcus	Reduced	Coronary artery disease	16S rRNA sequencing	Liu Z[1]	2019	Faeces	Human
	Elevated	Coronary artery disease	16S rRNA sequencing	Liu H[4]	2020	Faeces	Human
	Elevated	Atherosclerosis	16S rRNA sequencing	Jie Z[5]	2017	Faeces	Human
Enterococcus	Elevated	Coronary artery disease	16S rRNA sequencing	Zhu Q[2]	2018	Faeces	Human
	Elevated	Coronary artery disease	16S rRNA sequencing	Liu Z[1]	2019	Faeces	Human

References of Supplementary Table 1:

1. Liu Z, Li J, Liu H, Tang Y, Zhan Q, Lai W, et al. The intestinal microbiota associated with cardiac valve calcification differs from that of coronary artery disease. Atherosclerosis. 2019; 284: 121–8.

2. Zhu Q, Gao R, Zhang Y, Pan D, Zhu Y, Zhang X, et al. Dysbiosis signatures of gut microbiota in coronary artery disease. Physiol Genomics. 2018; 50: 893–903.

3. Toya T, Corban MT, Marrietta E, Horwath IE, Lerman LO, Murray JA, et al. Coronary artery disease is associated with an altered gut microbiome composition. PLoS One. 2020; 15: e0227147.

4. Liu H, Chen X, Hu X, Niu H, Tian R, Wang H, et al. Alterations in the gut microbiome and metabolism with coronary artery disease severity. Microbiome. 2019; 7: 68.

5. Jie Z, Xia H, Zhong SL, Feng Q, Li S, Liang S, et al. The gut microbiome in atherosclerotic cardiovascular disease. Nat Commun. 2017; 8: 845.

6. Emoto T, Yamashita T, Kobayashi T, Sasaki N, Hirota Y, Hayashi T, et al. Characterization of gut microbiota profiles in coronary artery disease patients using data mining analysis of terminal restriction fragment length polymorphism: gut microbiota could be a diagnostic marker of coronary artery disease. Heart Vessels. 2017; 32: 39–46.

7. Gargari G, Deon V, Taverniti V, Gardana C, Denina M, Riso P, et al. Evidence of dysbiosis in the intestinal microbial ecosystem of children and adolescents with primary hyperlipidemia and the potential role of regular hazelnut intake. FEMS Microbiol Ecol. 2018 May 1;94(5).

8. Zheng YY, Wu TT, Liu ZQ, Li A, Guo QQ, Ma YY, et al. Gut Microbiome-Based Diagnostic Model to Predict Coronary Artery Disease. J Agric Food Chem. 2020; 68: 3548–57.

9. Tan C, Wu Q, Wang H, Gao X, Xu R, Cui Z, et al. Dysbiosis of Gut Microbiota and Short-Chain Fatty Acids in Acute Ischemic Stroke and the Subsequent Risk for Poor Functional Outcomes. JPEN J Parenter Enteral Nutr. 2021; 45: 518–29.

10. Karlsson FH, Fåk F, Nookaew I, Tremaroli V, Fagerberg B, Petranovic D, et al. Symptomatic atherosclerosis is associated with an altered gut metagenome. Nat Commun. 2012; 3: 1245.

11. Kehrmann J, Menzel J, Saeedghalati M, Obeid R, Schulze C, Holzendorf V, et al. Gut Microbiota in Human Immunodeficiency Virus-Infected Individuals Linked to Coronary Heart Disease. J Infect Dis. 2019; 219: 497–508.

12. Zhang Y, Xu J, Wang X, Ren X, Liu Y. Changes of intestinal bacterial microbiota in coronary heart disease complicated with nonalcoholic fatty liver disease. BMC Genomics. 2019; 20: 862.
